# Delayed and Concurrent Stereotactic Radiosurgery in Immunotherapy-Naïve Melanoma Brain Metastases

**DOI:** 10.3390/cancers16223733

**Published:** 2024-11-05

**Authors:** Christine E. Hadley, Jennifer K. Matsui, Dukagjin M. Blakaj, Sasha Beyer, John C. Grecula, Arnab Chakravarti, Evan Thomas, Raju R. Raval, James B. Elder, Kyle Wu, Kari Kendra, Pierre Giglio, Joshua D. Palmer

**Affiliations:** 1 College of Medicine, The Ohio State University, Columbus, OH 43210, USA; 2 Department of Radiation Oncology, The Ohio State University Wexner Medical Center, Columbus, OH 43210, USA; 3 Department of Neurosurgery, The Ohio State University Wexner Medical Center, Columbus, OH 43210, USA; 4 Division of Neuro-Oncology, Department of Neurology, The Ohio State University Wexner Medical Center, Columbus, OH 43210, USA

**Keywords:** melanoma, brain metastases, immunotherapy, stereotactic radiosurgery

## Abstract

Melanoma remains a formidable challenge in oncology, causing the majority of skin cancer deaths in the United States, with brain metastases contributing substantially to this mortality. This paper reviews the current therapeutic strategies for melanoma brain metastases, with a focus on delayed and concurrent stereotactic radiosurgery (SRS). While surgery and traditional chemotherapy offer limited efficacy, recent advances in immunotherapy, particularly immune checkpoint inhibitors (ICIs), have played a major role in the advancement and improved efficacy of the treatment of cancers, including brain metastases. Recent studies indicate that monotherapy with ICIs may lead to a higher median overall survival compared to historical benchmarks, potentially allowing patients to delay radiosurgery. Other studies have found that combining SRS with ICIs demonstrates promise, with results indicating improved intracranial control. Ongoing clinical trials explore novel combinations of immunotherapies and radiotherapies, aiming to optimize treatment outcomes while minimizing adverse effects. As treatment options expand, future studies will be necessary to understand the interplay between therapies and their optimal sequencing to improve patient outcomes.

## Simple Summary

Melanoma brain metastases have historically been challenging to treat and have significant effects on the overall survival of these patients. This review article summarizes current treatment modalities including surgery, radiation therapy, and systemic therapies with a focus on immune checkpoint inhibitors. These various treatment options are often used in conjunction to have a greater therapeutic effect on patient outcomes, and this article especially illustrates the role of immunotherapies used alongside stereotactic radiosurgery, a form of radiotherapy.

## 1. Introduction

Melanoma has historically been difficult to treat, as many cases are not discovered until later in the progression of the disease or after the melanoma has metastasized, oftentimes to the brain [1,2]. Treatment options for brain metastases in the past were previously limited to surgery and chemotherapy; however, in recent decades, stereotactic radiosurgery (SRS) has become a powerful treatment modality that has improved intracranial disease control [3]. This is in addition to several immune checkpoint inhibitors (ICIs), with ipilimumab, nivolumab, and relatlimab being the therapies most commonly studied in recent clinical trials. Much of the research in this field is now being dedicated to determining how best to combine radiotherapy modalities and ICIs to maximize the therapeutic effect while keeping adverse effects such as drug toxicities and radiation necrosis to a minimum, as these are the most common causes treatment discontinuation. Many studies included in this review conclude that future research should increase the size of patient cohorts and should be framed prospectively to generate greater statistical power and stronger evidence for changing the current paradigm of treatment of melanoma brain metastases. 

## 2. Epidemiology of Metastatic Melanoma

Melanoma continues to be the deadliest form of skin cancer, accounting for 80% of all skin cancer deaths in the United States. When including all stages of melanoma, the 5-year survival rate is relatively high at 93.5%, but when extrapolated for metastatic melanoma, the 5-year survival rate drops to 35.1% [1]. In the United States, the lifetime risk of developing melanoma is now reported as 1 in 39 and 1 in 58 for men and women, respectively [2]. The primary risk factor for developing melanoma is UV exposure, but this risk factor can vary according to the individual’s genetics, melanin content, and UV exposure wavelength [4].

Patients with melanoma are frequently diagnosed at later stages due to asymptomatic initial presentation, with non-specific symptoms appearing later in the course of the disease [5]. Of new melanoma cases, 4% are diagnosed as metastatic upon presentation, most frequently spreading to the liver, bone, and brain [2]; brain metastases are most often the ultimate cause of death [6]. The median survival from the time of diagnosis of brain metastases is reported to be as low as 4 and up to 38 months depending upon treatment strategies, with improved survival likely due to the introduction of immune checkpoint inhibitors and targeted therapies [2,7]. Factors influencing survival also include age at diagnosis, performance status, and the number of total metastatic lesions [2,8].

## 3. Therapies for Brain Metastases in Melanoma

### 3.1. Surgery

The management of brain metastases continues to be influenced by the Patchell and colleagues’ trial, where patients with a single brain metastasis from an extracranial primary cancer who were treated with surgical resection and RT had better outcomes than patients treated with RT alone [9]. Historically, the role of surgery in the treatment of metastatic melanoma of the central nervous system (CNS) has been limited to local disease control and the palliation of neurologic symptoms caused by mass effects of larger tumors [10]. As our understanding of molecular drivers and the tumor microenvironment has evolved, the treatment strategies have developed a growing emphasis on targeted and immunomodulatory therapies. Current surgical strategies continue to focus on local control, obtaining diagnostic tissue, or addressing the acute and progressive neurologic decline refractory to steroids and other therapies [11]. It should also be noted that surgery is indicated in the case of hemorrhage of a CNS metastasis, which can be seen more often in melanoma brain metastases, specifically [12].

A retrospective study conducted in 2000 with 91 patients reported the median survival following resection to be 6.7 months, with no difference in survival between patients who received post-operative WBRT versus those who did not [13]. However, another retrospective study of 34 patients reports that surgical resection along with adjunctive WBRT yielded a statistically significant increase in the overall survival (*p* = 0.002) [14]. Multiple studies also reported that patients with solitary lesions tend to have better post-operative prognoses than patients with multiple resected lesions [13,15]. Recent data suggest that in cases of well-controlled systemic disease, surgical intervention may be used to optimally time and maximize the effectiveness of immune and radiation therapies [16]. 

Notably, there are primarily retrospective surgical studies for patients with melanoma brain metastases, which is understandable given the barriers to creating a prospective trial (e.g., ethical considerations). Available retrospective studies do support surgical resection, particularly in solitary lesions, and removal of such lesions may lead to improved survival outcomes [17].

### 3.2. Cytotoxic Chemotherapy

Chemotherapy has had limited success in treating intracranial metastatic melanoma, likely due to the inability to pass the blood–brain barrier (BBB) to an extent that would be therapeutic. Drugs such as Temozolomide and Fotemustine have been the most commonly used chemotherapeutic agents; however, neither has proven particularly effective in controlling the disease [18]. A 2004 study conducted using solely temozolomide reported only a 7% overall response rate and a median survival of 2.2 months [19]. Other studies with various combinations of chemotherapeutic agents also reported meager overall response rates ranging from 10 to 25% [20]. 

### 3.3. Radiation Treatment in Metastatic Melanoma

#### 3.3.1. Whole Brain Radiation Therapy

Historically, the method of external beam radiation therapy commonly used in the treatment of brain metastases was whole brain radiation therapy (WBRT), which as previously mentioned, was and still is often used in conjunction with systemic treatments as an adjuvant therapy [21]. Due to the neurocognitive side effects caused by WBRT, it is now often reserved as a palliative treatment after all other courses of treatment have been exhausted [18]. A single-institution study conducted in 2021 of 63 patients with metastatic brain disease and treated with WBRT showed an overall survival (OS) of 7 months [22]. The same study also reported that even patients who experienced adverse effects of the treatment did not have a statistically significant difference in the OS [22]. A 2020 study, however, provided conflicting results with 72 patients receiving WBRT who had no significant improvement in the OS [23]. This is in addition to a 2019 study including 215 patients who had local treatment of brain metastases via SRS or surgical resection who were then randomized to an observation group or a group receiving subsequent WBRT. Notably, the study concluded that adjuvant WBRT had no impact on intracranial control, survival or the preservation of performance status [24]. The discordant data for the efficacy of WBRT have provided an impetus for radiation oncologists to develop more focused and effective radiological techniques such as SRS.

#### 3.3.2. Stereotactic Radiosurgery

Currently, SRS plays a critical role in the management of brain metastases; utilization of SRS has continued to rise as systemic treatments have improved [25]. SRS is also a favorable therapeutic option, as there is no need to delay systemic therapy being received at the time, and SRS can be completed in as little as a single session or up to five sessions [26]. This is opposed to WBRT, which is typically delivered in 10–15 fractions over 2–3 weeks, though WBRT can be reserved for the patients who are not good candidates for focal treatment [27]. Additionally, SRS has also been shown to have no effect on the likelihood of subsequent hemorrhage [28].

Even before immunotherapy, SRS had been used as monotherapy in patients with singular, smaller intracranial lesions due to the more specific targeting of radiation [29,30]. Earlier detection of intracranial metastatic lesions via MRI has also allowed SRS to be more widely utilized, as it tends to have more favorable results on smaller lesions discovered earlier in the disease progression [31]. This is further demonstrated by a meta-analysis of the tumor control probability of various-sized tumors after SRS or fractionated SRS (fSRS) [32]. For small tumors (diameter ≤ 20 mm), single-dose fractions 24 Gy yielded a 1-year local control rate of 95%. Tumors 21–30 mm in diameter treated with a single 18 Gy fraction had a 75% 1-year local control rate, and tumors 31–40 mm treated with a 15 Gy fraction had a 65% 1-year local control. However, this study showed that larger tumors (21–40 mm) treated with 3–5 fractions of 27–35 Gy had 80% 1-year local control, suggesting that multiple fractions, as opposed to a single fraction, could be beneficial in treating larger lesions [32].

### 3.4. Targeted Therapy in Metastatic Melanoma

The most common targeted therapy (TT) in the treatment of metastatic melanoma is BRAF and MEK inhibition. These treatments have been proven to be effective, but it should be noted that even patients who have achieved complete remission (CR) have a higher risk of recurrence if TT is discontinued, as evidenced by multiple studies [33,34]. However, one study also concluded that patients who received TT for longer than 16 months did not have a shorter overall progression-free survival time compared to patients who received ongoing targeted therapy, suggesting that the longer the patient has undergone TT, the more protective the treatment becomes even if discontinued [33]. Though early cessation of the treatment puts patients at risk for subsequent disease progression, it appears that patients are responsive to a rechallenge with BRAF and/or MEK inhibitors [34].

#### 3.4.1. Interleukin-2

As systemic targeted therapies advanced, interleukin-2 (IL-2) became an increasingly interesting modality of metastatic treatment by leveraging its antitumor properties [35]. It is proposed that IL-2, among other cytokines, incites an inflammatory response that promotes the action of CD-8 T-cells and has enhanced antitumor genetic properties in animal models [36]. Although high-dose IL-2 was the first immunotherapy to provide meaningful efficacy, only a minority of the patients demonstrated significant overall objective response rates, and there was associated multi-organ toxicity [37,38] .

#### 3.4.2. Immunotherapy Checkpoint Inhibitors

Immune checkpoint inhibitors (ICIs) have become the standard of care for aggressive cancers, such as metastatic melanoma, and have been shown to improve survival [39]. These treatments are thought to enhance the body’s defensive response by working at various immune checkpoints to decrease the cancer cell’s ability to evade the immune response. Ipilimumab interacts with the inhibitory cytotoxic T-lymphocyte-associated protein 4 (CTLA-4) at the cell surface, thereby releasing the inhibition of the immune response and effectively exposing the cancer cells to attack. Pembrolizumab and nivolumab are PD-1 inhibitors that work in a similar mechanism to CTLA-4 inhibitors by inhibiting a cell surface receptor that would otherwise prevent T-cells from attacking the cancerous cells. A list of completed clinical trials assessing the efficacy and safety of ICIs is outlined in Table 1.

## 4. Combination Immunotherapy

Early in the evolution of immunotherapy, a combination of CTLA-4 and PD-1 inhibitors has proven to be more effective than monotherapy with either treatment method [47]. Thus, virtually all studies involving the treatment of metastatic melanoma in the most recent decade combine various treatment modalities (e.g., multiple TT drugs, multiple ICIs, or a combination of TT and ICIs) [48]. 

Most treatment regimens for metastatic melanoma now combine TT and IT in an effort to maximize the OS. Though it can be tempting for physicians to add on more therapies as they become available, one study examined the OS of patients who had previously received a BRAF/MEK inhibitor and then received either anti-PD1 (PD1) or PD1 and anti-CTLA-4 (ipilimumab) and found that there was no significant difference in the OS between the groups [49]. The study did note that the PD1 plus ipilimumab group did experience more severe side effects while seeing little to no improvement in the OS [49]. Interestingly, a 2021 study evaluated 23 BRAF-mutated patients who received PD1 treatment and then received BRAF and/or MEK inhibitor therapy as their second-line treatment and found that 55% of these patients were responsive with manageable levels of toxicity [50]. However, a more robust 2022 study of 454 BRAF-mutated patients found that participants showed statistically significant improvements in the OS and overall progression-free survival (PFS) when treated initially with TT rather than the group treated initially with ICIs [51]. 

Looking more specifically at melanoma patients with asymptomatic brain metastases, PD1 ICIs are the most common treatment modality in advanced melanoma [52]. Patients with symptomatic brain metastases are still more likely to receive surgery as the first course of treatment [53]. Though multiple studies concluded that PD1 is effective in treating brain metastases, care needs to be taken to perform CNS imaging throughout the treatment to monitor intracranial disease progression [52,54]. It should also be noted that it appears PD1 treatment has a durable and complete response even after discontinuation [52]. Some studies have also shown that PD1 in combination with ipilimumab can be effective in achieving intracranial response in asymptomatic, untreated brain metastases [47,55]. One 2019 study reports that PD1 and ipilimumab can be used in patients with symptomatic brain metastases, but these patients have a lower response rate [56]. Another confounding variable that has been studied more recently is the effect of corticosteroids, which are administered to control the symptomatic patients. Administration of corticosteroids has been shown to decrease the intracranial response rate of ipilimumab treatment and TT [57,58].

### Nivolumab/Ipilimumab Combined Therapy

A phase III randomized study of 945 patients compared nivolumab alone, nivolumab plus ipilimumab, and ipilimumab alone in patients with untreated metastatic melanoma [47]. The primary end points were PFS and OS, with secondary endpoints including objective response rate, tumor PD-L1 expression as a predictive biomarker, and safety. Median follow-up ranged from 12.2 to 12.5 months. Discontinuation related to disease progression occurred in 49.2% and 65.0% in the nivolumab and ipilimumab monotherapy groups, respectively. The most common reason for discontinuation in the nivolumab plus ipilimumab group was the toxic effects of the study drug (38.3%). Common grade 3 or 4 adverse events were diarrhea, colitis, and increased alanine aminotransferase level. Notably, there was longer PFS in the nivolumab plus ipilimumab group than the ipilimumab alone group (hazard ratio for death or disease progression, 0.42; CI 95% 0.43–0.76; *p* < 0.001). The median PFS with the combination therapy was comparable to the reports with combination BRAF and MEK inhibitors in patients with BRAF-mutated metastatic melanoma [47,59].

Recently, long-term outcomes were reported for CheckMate 067 [60]. Wolchok et al. reported a median OS of 72.1 months in the combination group compared to 36.9 and 19.9 months in the nivolumab and ipilimumab alone groups, respectively, with a minimum follow-up of 6.5 years. The median PFS was 11.5 months (95% CI 5.1–10.2 months) in the combination group compared to 6.9 months (95% CI 5.1–10.2 months) and 2.9 months (95% CI 2.8–3.2 months) in the nivolumab and ipilimumab groups, respectively. The long-term survival analysis found improved OS with nivolumab alone or in combination with ipilimumab compared to ipilimumab alone. In a subgroup analysis comparing patients with or without baseline liver metastases, the authors reported worse survival outcomes in patients with baseline liver metastases.

Additionally, CheckMate 204 compared therapy with a combination of nivolumab and ipilimumab in both symptomatic and asymptomatic patients with brain metastases, with 54% and 17% objective responses in asymptomatic patients and symptomatic patients, respectively [40]. However, these results were accompanied by an adverse event (grades 3 and 4) rate of 55% in a 101-patient cohort. In another study, 54% of the patients in the cohort treated with both nivolumab and ipilimumab also experienced grade 3 and 4 adverse events [55].

## 5. Concurrent Stereotactic Radiosurgery and Immune Checkpoint Therapy

Many studies have also reported the benefits of combining SRS with checkpoint therapies while taking into consideration the subsequent toxicities. An 80-patient study reported a 42% and 17% 12-month PFS in patients with melanoma brain metastases treated with SRS and subsequent nivolumab or ipilimumab, respectively, and also showed that multi-fraction SRS improved PFS significantly more than single-fraction SRS [61]. Compared to the studies that examined the combined effects of nivolumab plus ipilimumab, grade 3 adverse events in this study were reduced, with only 24% and 17% of patients treated with SRS plus ipilimumab or nivolumab, respectively, experiencing toxicity. 

Another study of 28 patients similarly compared the local control of melanoma brain metastases in patients treated with SRS alone or SRS in combination with various other immunotherapies and targeted therapies (administered up to 4 weeks before or after SRS) [62]. The one-year local control rate in patients treated with SRS plus concurrent immunotherapy or targeted therapy was 100% in this study as compared to 83% in the patients receiving SRS plus non-concurrent immunotherapy or targeted therapy (*p* = 0.023), suggesting the timing of the targeted therapies being a key player in the efficacy of the treatment regimen. Regarding the adverse effects, investigators noted that the nature of the study did not allow for a thorough analysis of adverse effects but did report that there was no statistically significant difference in the rates of radiation necrosis. Though the data suggest significant results, further larger, prospective studies would bolster these conclusions. 

A study by Bodensohn et al. specifically examined the adverse effects of the treatment of melanoma brain metastases with SRS plus ipilimumab and nivolumab. They concluded that SRS either 7 days before or after the treatment with combined nivolumab and ipilimumab resulted in significantly greater rates of grade 3 adverse effects [63]. Though the patient cohort (31 patients) was relatively smaller than the previous studies mentioned, these results warrant further consideration on the optimal timing of treatments. 

Another study of 10 patients with metastatic melanoma also demonstrates the described “immune-priming” effect of RT even after the brain metastases developed resistance to ICIs [64]. The proposed mechanism is that RT increases inflammatory cytokines within the tumor and primes the environment for ICIs, but more research needs to be conducted on this phenomenon with a more robust pool of participants [64]. Though this study showed the benefits of subsequent RT, a meta-analysis of concurrent SRS and ICI therapy by Lehrer et al. argues that concurrent treatment significantly improves the overall survival in patients with brain metastases [65]. These results were supported by a 143-patient retrospective study that also found the highest overall response rate in patients treated with both RT and ICIs [66]. The same study with a subgroup of 41 patients also showed responsiveness to RT even after intracranial disease progression while on ICIs [66].

From a mechanistic perspective, the concurrent use of RT with ICIs may have synergistic effects, where RT may potentiate the effects of ICIs [67]. As described by Weichselbaum et al., RT has the ability to create an inflammatory microenvironment and enhance the innate and adaptative immune responses against tumors [68]. Further investigations are necessary to determine the optimal dose and fractionation when utilizing RT concurrently with ICIs. Additionally, as seen in the study by Bodensohn, there is the risk of increased toxicity with combined therapy, and certain biomarkers may be useful in predicting the response to therapy [69].

## 6. Ongoing Clinical Trials

Table 2 includes ongoing clinical trials assessing the efficacy of SRS and ICIs. A phase III study is currently in the recruiting phase and aims to better assess the benefits of SRS plus targeted or immunotherapies versus targeted or immunotherapies alone in asymptomatic patients with melanoma brain metastases, primarily examining PFS in these groups [70]. The study plan will include patients who receive many different forms of systemic therapies, which could perhaps allow for a greater participant pool than studies that have only examined the effects of combined nivolumab and ipilimumab. The study will also aim to answer the question of when to administer treatment to maximize treatment efficacy while avoiding adverse effects. 

Another phase I/II study, which is not yet recruiting, will be studying a novel immunotherapy combination of bevacizumab plus combined nivolumab plus ipilimumab in addition to hypofractionated stereotactic radiotherapy (hSRT) in patients with symptomatic melanoma brain metastases, with the primary goal of examining the safety of this treatment schedule [71]. The results of this study could also provide more insight on best practices for the timing of treatments, as it specifically outlines that patients will receive hSRT between the first and second rounds of combined nivolumab and ipilimumab therapy. Other secondary outcomes include PFS, OS, overall response rate, intercranial clinical benefit, and the magnitude of steroid use reduction. 

Additionally, a phase II study already underway is aimed at studying the combination of nivolumab with relatlimab on melanoma brain metastases [72]. The primary goals of the study are to determine the efficacy of this novel therapy combination while also determining the safety of this regimen. This clinical trial was likely prompted by the high toxicity experienced by many patients treated with the combination of nivolumab plus ipilimumab and the need to explore other combinations as new immune checkpoint inhibitors, such as relatlimab, are approved. Recent data show that relatlimab plus nivolumab and ipilimumab plus nivolumab have comparable overall response rates, but the combination with relatlimab results in fewer adverse effects. More data, however, are still needed in order to determine more long-term effects of this new combination, and the effects of increased or decreased PD-L1 expression must also be considered [72]. 

## 7. Delayed Radiation Therapy 

SRS has been shown to be an effective treatment modality in brain metastases and has now been studied with concurrent ICI treatment. Though recent studies have suggested RT could be delayed with ICIs, Liermann et al. argue that the treatment of brain metastases with SRS in conjunction with ICIs is safe and effective and could potentially have lower toxicity [73]. 

A meta-analysis by Rulli et al. aimed to compare the progression-free survival rate, overall response, and objective response rate at the extracranial and intracranial sites of various treatment methods, such as targeted therapy, combined immunotherapy, and monoimmunotherapy, with or without radiotherapy. Though their analyses showed that combined immunotherapy had superior progression-free survival and overall survival compared to immunotherapy plus radiotherapy, it is important to note that only two studies included in the meta-analysis treated patients with combined immunotherapy plus radiotherapy [74]. These findings draw attention to the need for more prospective research to be conducted to establish more firm guidelines on the optimal utilization of SRS in combination with immunotherapies, which have become heavily utilized therapeutic agents. The limited data available also require more inquiry into optimal timing if SRS is to be used at all in these patients. 

## 8. Conclusions

Historically, the prognosis of metastatic melanoma was extremely poor, but in recent decades, survival outcomes have improved with the introduction of ICIs. This is especially true for patients with brain metastases, whose median OS without treatment can be as few as four months. With the rising popularity of ICIs for the treatment of melanoma brain metastases, the role of SRS is evolving. Studies have suggested that monotherapy has resulted in increased median OS compared to historical controls. The concurrent use of SRS with ICIs has also been explored and may impart a synergistic effect and lead to improved intracranial control. As new targeted therapies continue to emerge for the treatment of metastatic melanoma, it is critical to identify which patients may benefit from delayed or concurrent SRS.

## Figures and Tables

**Table 1:** Clinical trials evaluating targeted therapies in melanoma brain metastases.

Reference	Study Type	Treatment Group(s)	Details	Median PFS	Median OS	Median Follow-Up (Months)	Other Results
Tawbi et al. [40]	Phase 2 (CheckMate 204)	Cohort A: Asymptomatic MBM	Combination nivolumab plus ipilimumab	IC PFS at 36 months was 54.1% (95% CI 42.7–64.1).	At 36 months was 71.9% (95% CI 61.8–79.8).	34.3	
		Cohort B: Symptomatic MBM	Combination nivolumab plus ipilimumab	IC PFS at 36 months was 18.9% (95% CI 4.6–40.5).	At 36 months was 36.6% (95% CI 14.0–59.8).	7.5	
Long et al. [41]	Phase 2 (ABC)	Cohort A: Asymptomatic MBM with no prior local therapy	Ipilimumab + Nivolumab	12 and 24 mo IC PFS were 49% and 49%, respectively.	12 and 24 mo OS were 63% and 63%, respectively.	34	TRAEs grade 3/4 toxicity was 54%.
		Cohort B: Asymptomatic MBM with no prior local therapy	Nivolumab	12 and 24 mo IC PFS were 20% and 15%, respectively.	12 and 24 mo OS were 60% and 51%, respectively.		TRAEs grade 3/4 toxicity was 20%.
		Cohort C: Patients who failed local therapy	Nivolumab	12 and 24 mo IC PFS were 6% and 6%, respectively.	12 and 24 mo OS were 31% and 19%, respectively.		TRAEs grade 3/4 toxicity was 13%.
Long et al. [42]	Phase 2 (BREAK-MB)	Cohort A: No previous local treatment for MBM	Dabrafenib	Median PFS 8.1 (3.1–16.1)	Median OS, 16.3 (6.9–22.4)		TRAEs grade 4 toxicity was 4%.
		Cohort B: Progressive MBM after local treatment	Dabrafenib	Median PFS, 15.9 (7.9–22.4)	Median OS, 21.9 (15.3–NR)		TRAEs grade 4 toxicity was 2%.
Davies et al. [43]	Phase 2 (COMBI-MB)	Cohort A: BRAFV600E-positive, asymptomatic MBM, with no previous local therapy, and an ECOG of 0 or 1	Dabrafenib plus trametinib	Median PFS, 5.6 (5.3–7.4)	Median OS, 10.8 (8.7–19.6)	8.5	
		Cohort B: BRAFV600E-positive, asymptomatic MBM, with previous local therapy, and an ECOG of 0 or 1.	Dabrafenib plus trametinib	Median PFS, 7.2 (4.7–14.6)	Median OS, 24.3 (7.9–NR)		
		Cohort C: BRAFV600D/K/R-positive, asymptomatic MBM, with or without previous local therapy, and an ECOG of 0 or 1.	Dabrafenib plus trametinib	Median PFS, 4.2 (1.7–6.5)	Median OS, 10.1 (4.6–17.6)		
		Cohort D: BRAFV600D/E/K/R-positive, symptomatic MBM, with or without previous local therapy, and an ECOG of 0, 1, or 2.	Dabrafenib plus trametinib	Median PFS, 5.5 (2.8–7.3)	Median OS, 11.5 (6.8–22.4)		
McArthur et al. [44]	Phase 2	Cohort 1: Previously untreated MBM, BRAFV600 mutated	Vemurafenib	Median PFS, 3.7 months (range, 0.03–33.4; IQR 1.9–5.6)	Median OS, 8.9 months (range 0.6–34.5; IQR 4.9–17.0)	9.6 (95% CI 7.2–11.5)	TRAEs grade 3/4 in 59 (66%).
		Cohort 2: Previously treated MBM, BRAFV600 mutated		Median PFS, 4.0 months (range, 0.3–27.4; IQR 2.2–7.4)	Median OS, 9.6 months (range 0.7–34.3; IQR 4.5–18.4)		TRAEs grade 3/4 in 36 (64%).
Kluger et al. [45]	Phase 2	One or more asymptomatic, untreated 5 to 20 mm MBM not requiring corticosteroids	Pembrolizumab	Median PFS, 2 months	Median OS, 17 months	34	TRAEs grade 3/4 in 36 (64%).
Di Giacomo et al. [46]	Phase 3 (NIBIT-M2)	Group 1: Fotemustine		Median PFS, 3.0 (95% CI 2.3–3.6)	Median OS was 8.5 months. (95% CI 4.8–12.2)		TRAEs grade 3/4 in 11 (48%).
		Group 2: Ipilimumab plus fotemustine		Median PFS, 3.3 (95% CI 1.2–5.4)	Median OS, 8.2 months (95% CI, 2.2–14.3)		TRAEs grade 3/4 in 18 (69%).
		Group 3: Ipilimumab plus nivolumab		Median PFS, 8.7 (95% CI 0.0–19.9)	Median OS, 29.2 months (95% CI, 0–65.1)		TRAEs grade 3/4 in 8 (30%).

Abbreviations: MBM = melanoma brain metastases; PFS = progression-free survival; OS = overall survival; TRAEs = treatment-related adverse events.

**Table 2:** Ongoing clinical trials assessing safety and efficacy of stereotactic radiosurgery and immune checkpoint inhibitor therapies.

Study	Phase	Status	Title	ICIs	Description	Primary outcomes
NCT05522660	3	Recruiting	Immunotherapy or Targeted Therapy With or Without SRS for Patients With MBM or NSCLC	anti-PD1/L1 ± Ipilimumab	ICIs plus SRS vs. ICIs alone for MBM.	CNS-specific PFS
NCT06163820	½	Not yet recruiting	Bevacizumab and ICIs + hSRT in Symptomatic Melanoma Brain Metastases (BETTER)	Nivolumab + Ipilimumab	Symptomatic MBM evaluating safety and efficacy of combination bevacizumab, Nivo + Ipi, and hSRT.	DLT
NCT05341349	1	Recruiting	Stereotactic Radiosurgery and Immune Checkpoint Inhibitors With NovoTTF-100M for the Treatment of Melanoma Brain Metastases	Pembrolizumab	Side effects and possible benefit of SRS and ICIs with NovoTTF-100 M for the treatment of MBM.	Percent of grade 3 CNS toxicity
NCT05669352	½	Not yet recruiting	A Study of Oral IRAK-4 Inhibitor CA-4948 in Combination With Pembrolizumab Following SRS in Patients With MBM	Pembrolizumab	IRAK-4 inhibitor CA-4948 in combination with Pembro following SRS in patients with MBM.	IC intervention 1 year after initial SRS
NCT05703269	3	Recruiting	Comparing Single vs. Multiple Dose Radiation for Cancer Patients With Brain Metastasis and Receiving Immunotherapy (HYPOGRYPHE)	anti-PD1/L1 ± CTLA-4 agent	Compare SSRS vs. FSRS in patients with brain metastases (including MBM) on ICI therapy.	Grade 2+ ARE
NCT04074096	2	Recruiting	Binimetinib Encorafenib Pembrolizumab +/− SRS in BRAFV600 MBM (BEPCOME-MB)	Pembrolizumab	Assess the tolerability of binimetinib–encorafenib–pembrolizumab combination therapy +/− SRS.	IC PFS

Abbreviations: MBM = melanoma brain metastases; NSCLC = non-small-cell lung cancer; ICIs = immune checkpoint inhibitors; PFS = progression-free survival; Nivo = nivolumab; Ipi = ipilimumab; DLT = dose-limiting toxicities; hSRT = hypofractionated stereotactic radiotherapy; Pembro = pembrolizumab; SRS = stereotactic radiosurgery; SSRS = single fraction stereotactic radiosurgery; FSRS = fractionated stereotactic radiosurgery; ARE = adverse radiation effect; IC = intracranial.
